# Pulmonary benign metastasizing leiomyoma presented as acute respiratory distress

**DOI:** 10.1002/rcr2.216

**Published:** 2017-01-19

**Authors:** Jean Pastré, Karine Juvin, Bertrand Grand, Laure Gibault, Judith Valcke, Dominique Israël‐Biet

**Affiliations:** ^1^ Université Paris DescartesSorbonne Paris Cité and AP‐HPParisFrance; ^2^ Service de PneumologieHôpital Européen Georges PompidouParisFrance; ^3^ Service de Chirurgie ThoraciqueHôpital Européen Georges PompidouParisFrance; ^4^ Service d'Anatomo‐pathologieHôpital Européen Georges PompidouParisFrance

**Keywords:** Benign metastasizing leiomyoma, lung, metastases, pleura

## Abstract

Benign metastasizing leiomyoma (BML) is a very rare condition and is characterized by the presence of benign smooth muscle tumours in organs distant from the uterus, most commonly the lung. It generally affects women of reproductive age and prognostic is usually excellent. However, the course of the disease is unpredictable. We report here the case of a 76‐year‐old woman with a previous medical history of uterine benign leiomyomas in whom BML was acutely revealed by a respiratory distress due to voluminous pulmonary and pleural leiomyomas requiring surgical extraction. Clinical evolution was remarkable by resistance to medical treatment and development of rare bone localization.

BML is a contradictory entity characterized by benign histological features but with metastatic potential. Pulmonologists as well as oncologists in charge of patients with multiple pulmonary nodules and a history of uterine leiomyoma should be aware of this potential diagnosis in order to implement appropriate diagnostic procedures for this benign tumour.

## Introduction

Benign metastasizing leiomyoma (BML), characterized by the presence of benign smooth muscle tumours in organs distant from the uterus, most commonly the lung, is a very rare condition (<200 cases reported in the literature). It generally affects women of reproductive age with a history of benign uterine leiomyoma resection 1. Pulmonary presentation is predominantly indolent with nodules incidentally discovered on routine chest X‐ray.

We report here the case of a 76‐year‐old woman in whom BML was acutely revealed by a respiratory distress and whose evolutive pattern was remarkable by resistance to medical treatment and development of a rare bone BML localization.

## Case Report

A 76‐year‐old woman was admitted in intensive care for acute respiratory distress. She complained of an increasing and isolated dyspnoea developed over the previous months. Her medical history included heavy smoking habits (75 pack‐years, given up 6 years prior to admission), ischaemic coronaropathy, and a total hysterectomy 4 years previously for postmenopausal metrorrhagia. It had revealed multiple subserosal uterine benign leiomyomas with no evidence of extrauterine involvement at that time.

**Figure 1:**
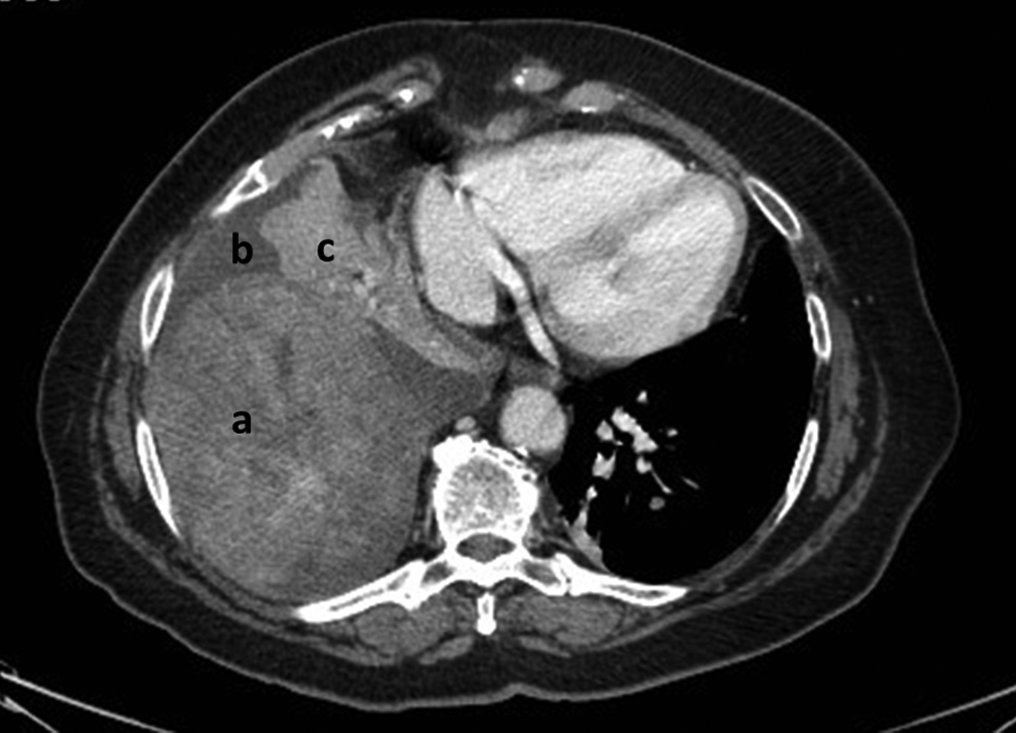
Initial computed tomography (CT) scan showing: a, large rounded pulmonary benign leiomyoma; b, pleural effusion; c, passive lung atelectasis.

On admission, she was severely hypoxemic with a saturation of 91% when breathing 6 L/mn of oxygen. Chest X‐ray showed a right, unilateral massive pleural effusion associated with bilateral parenchymal nodules. Biology was unremarkable. A pleural chest tube was rapidly inserted disclosing an exudate (49 g/L of proteins) with 80% lymphocytes. Clinical improvement was dramatic and chest high‐resolution computed tomography (HRCT) confirmed the presence of bilateral, well‐defined lung nodules with one of them reaching 15 cm in diameter (Fig. 1). Fiberoptic bronchoscopy disclosed an important extrinsic compression with no other abnormality.

**Figure 2:**
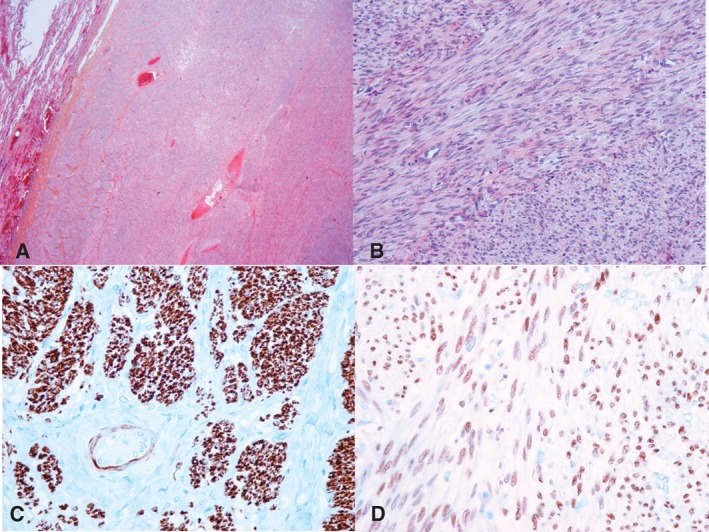
Benign metastatic leiomyoma (BML): (A) Hematoxylin eosin saffron (HES) stain ×20, BML nodule (right) compressing the residual pulmonary parenchyma (left). (B) HES stain ×200, fascicules of spindle cells without any cytonuclear atypia and few mitosis. (C) Diffuse expression of desmin by the tumour cells (×200). (D) Diffuse expression of oestrogen receptors (×200).

Thoracic surgery was indicated for 1 histological diagnosis and 2 control of lung external compression. Four bulky, purplish red, soft, and smooth tumours were excised from the right upper and lower lobes. Microscopic examination (Fig. 2) demonstrated fascicles of eosinophilic spindle cells with bland nuclei and inconspicuous mitotic activity (less than one mitotic figure per 10 high‐power fields). The cells were strongly positive for smooth muscle actin (SMA), desmin, and caldesmon as well as for oestrogen and progesterone receptors. No necrotic zone was observed. The initial uterine lesion was reviewed and diagnosis of leiomyoma confirmed, with no histological feature suggestive of malignancy, excluding the hypothesis of a metastatic leiomyosarcoma and suggesting the diagnosis of BML.

During follow‐up, the remaining pulmonary nodules slowly but markedly increased in size and respiratory symptoms recurred. Aromatase inhibitors were started to control their growth but patient's respiratory condition continued to worsen. Another surgery was performed to excise three pulmonary nodules. All of them displayed histological characteristics similar to those of the nodules previously removed. Pulmonary condition was then stabilized but 9 months later the patient was diagnosed with a new BML localization as an osteolytic right trochanteric lesion. Surgery was undertaken to ensure a preventive osteosynthesis. Again, pathological examination revealed characteristic BML features.

After a follow‐up of 45 months, our patient is presently doing well with no pulmonary symptom and no new lesion.

## Discussion

Benign metastasizing leiomyoma is a very rare condition mainly occurring in women of reproductive age with a history of uterine leiomyoma resected 1 month to 20 years previously 1, 2. Made of benign spindle‐shaped smooth muscle cells, BML has a metastatic potential to distant sites, most commonly lung but also to skin, lymph nodes, muscles, retroperitoneum, central nervous system, heart, and bones 3. The usual chest imaging of lung BML is unilateral (very rarely) or bilateral nodules of varying sizes, ranging from millimetric micronodules to voluminous lesions usually well circumscribed. Cystic or cavitary lesions have also been reported. In addition, endobronchial or endovascular localizations have also been described.

BML usually has a limited growth capacity and evolves silently with no pulmonary symptoms and a very limited impact on lung function. Histological proof is required for these tumours which often mimic lung cancer or more rarely benign conditions (tuberculoma, sarcoidosis, pneumoconiosis, lung collagen vascular diseases). Surgical biopsy is therefore the most frequent approach to histopathological confirmation of BML. Various immunohistochemical markers such as desmin, muscle‐specific actin, and caldesmon confirm smooth muscle differentiation. Presence of oestrogen and progesterone receptors suggests a female genital tract origin and a hormone‐dependent growth. The usually low expression of Ki 67 and absence of cytological atypia argues for their benign nature 1, 2. Altogether, our case was histologically quite typical with interlacing fascicles of smooth muscle cells without anaplasia or vascular invasion and a very low mitotic activity.

In several other aspects, our patient was quite atypical. First, her age was higher than usually reported in the literature. Second, clinical presentation was unusually acute due to a massive pleural effusion and large pleural and pulmonary nodules mimicking a metastatic lung cancer in this ex‐heavy smoker. Third, bone localization with lytic femoral BML lesion has to the best of our knowledge not been reported before.

Treatment options for BML include surgical removal in the first place, potentially iterative in case of recurring symptomatic lung nodules 1. On the other hand and because BML expresses oestrogen and progesterone receptors, oestrogen level control is a potential target. For this purpose, hysterectomy, oophorectomy, and long‐term hormone therapy, based upon gonadotrophin‐releasing hormone agonists, progesterone or aromatase inhibitors 4, 5, might be indicated to block hormone release and tumour growth. In our patient, pulmonary and pleural tumours continued to expand despite her postmenopausal age and aromatase inhibitors.

BML is a contradictory entity characterized by benign histological features but a highly metastatic potential. Pneumologists as well as oncologists in charge of patients with multiple pulmonary nodules and a history of uterine leiomyoma should be aware of this potential diagnosis in order to implement appropriate diagnostic procedures for this benign tumour.

## Disclosure Statements

No conflict of interest declared.

Appropriate written informed consent was obtained for publication of this case report and accompanying images.
